# Manual of British Botany, Containing the Flowering Plants and Ferns, Arranged According to the Natural Orders

**Published:** 1844-01

**Authors:** 


Art. IX.—*Manual of British Botany, containing the Flowering Plants and Ferns, arranged according to the Natural Orders.* By Charles C. Babington, m.a. f.l.s. f.g.s. &c.—*London*, 1843. 12mo, pp. 400.

We cannot better set before our readers the peculiar characters of this admirable little volume, than by quoting from the preface the author's own account of the motives for its production :

"From the attention which has long been paid to the elucidation of the Flora of Britain, and the numerous excellent botanists who have, since the time of the justly celebrated Ray, (not to go further back,) employed their talents upon an endeavour to determine the indigenous products of these kingdoms; the author, in common, it is believed, with most English botanists, did not suppose that much remained to be done in British botany; for he could not expect that, after the labours of such men as Smith, Hooker, Lindley, and others, and the publication of so invaluable and unrivalled a collection of figures as is contained in the English botany, there could still be many questions concerning the nomenclature, or any considerable number of unascertained species, the determination of which would fall to his lot. He had not however advanced far in the critical examination of our native plants, before he found that a careful comparison of indigenous specimens with the works of eminent continental authors, and with plants obtained from other parts of Europe, must necessarily be made; for it appeared, that in very many cases, the nomenclature employed in England was different from that used in other countries, that often plants considered as varieties were held to be distinct species abroad, that several of our species were only looked upon as varieties by them, and also that the mode of grouping into genera was frequently essentially different." (Preface, p. 5.)

Mr. Babington then traces these discrepancies to their true source—the *exclusive system*, which, arising in some degree from our insular position, has been cherished, during the early part of the present century, by the separation induced by the continual state of warfare between Britain and the continent; and which has prolonged the attachment to the Linnæan system, and the valuable, but frequently erroneous works founded upon it, to the almost total neglect of the works of continental authors, who, it must be remembered, have under their observation a very large proportion of the plants found in this country, and who have frequently great advantages over British botanists in studying the influence of circumstances in producing variations. In the preparation of the present work, the author states, with his characteristic modesty, that

"He has carefully examined nearly all the best European floras, comparing our plants with the descriptions contained in them, and in very many cases with foreign specimens of undoubted authenticity. In the adoption of genera and species, an endeavour has been made, by the examination of the plants themselves, to determine what are to be considered as truly distinct; thus, it is hoped, taking nature as a guide, and not depending upon the authority of any name, however distinguished. Still, let it not be supposed that any claim is made to peculiar accuracy, nor that the author considers himself qualified to dictate to any student of botany; for he is well aware that there are many points, upon which persons who have carefully studied the subject, may form different conclusions from those to which he has been led." (Preface, p. vii.)

To the faithful execution of this purpose, Mr. Babington's well-known character as a scientific botanist, is a sufficient guarantee ; and in regard to the typographical beauty and accuracy of the work, it is enough to say, that it is published by Mr. Van Voorst, and is worthy of his reputation.

